# Fibroblast Activation Protein-α as a Target in the Bench-to-Bedside Diagnosis and Treatment of Tumors: A Narrative Review

**DOI:** 10.3389/fonc.2021.648187

**Published:** 2021-08-19

**Authors:** Lei Xin, Jinfang Gao, Ziliang Zheng, Yiyou Chen, Shuxin Lv, Zhikai Zhao, Chunhai Yu, Xiaotang Yang, Ruiping Zhang

**Affiliations:** ^1^Department of Radiology, Affiliated Tumor Hospital of Shanxi Medical University, Taiyuan, China; ^2^Third Hospital of Shanxi Medical University, Shanxi Bethune Hospital, Shanxi Academy of Medical Sciences, Taiyuan, China; ^3^Department of Biochemistry and Molecular Biology, Shanxi Medical University, Taiyuan, China

**Keywords:** cancer-associated fibroblast, FAP substrates, immunosuppressive, targeted therapy, nanomaterials, fibroblast activation protein-α

## Abstract

Fibroblast activation protein-α (FAP) is a type II integral serine protease that is specifically expressed by activated fibroblasts. Cancer-associated fibroblasts (CAFs) in the tumor stroma have an abundant and stable expression of FAP, which plays an important role in promoting tumor growth, invasion, metastasis, and immunosuppression. For example, in females with a high incidence of breast cancer, CAFs account for 50–70% of the cells in the tumor’s microenvironment. CAF overexpression of FAP promotes tumor development and metastasis by influencing extracellular matrix remodeling, intracellular signaling, angiogenesis, epithelial-to-mesenchymal transition, and immunosuppression. This review discusses the basic biological characteristics of FAP and its applications in the diagnosis and treatment of various cancers. We review the emerging basic and clinical research data regarding the use of nanomaterials that target FAP.

## Introduction

In 1986, Rettig et al. (1) discovered fibroblast activation protein-α (FAP) using a monoclonal antibody (F19), which reacted with activated fibroblasts *in vitro*. Rettig initially described the FAP protein as a cell surface antigen expressed on epithelial cancer cells, most of the soft tissue sarcomas, granulation tissue, and some fetal mesenchymal fibroblasts. However, it was not expressed on normal fibroblasts or benign/malignant epithelial tumor cells, hence it was referred to as the “fibroblast activation protein”. Aoyama et al. (2) later discovered a 170 kDa membrane-bound gelatinase dimer at the invasive front of the human melanoma cell line LOX, which was named “seprase” due to its surface expression by Monsky et al. (3), although protein sequence analysis subsequently revealed that FAP and seprase were the same protein (4, 5). Busek et al. (6) later reported that generally FAP is not expressed in healthy adult mammalian tissues, although some FAP^+^ cells are present in the placenta and uterine stroma, particularly during the proliferative phase (7), embryonic tissue (8) and multipotent bone marrow stromal cells (9). Moreover, small amounts of FAP are present in plasma from humans and other mammals (approximately 100 ng/mL or 0.6 nmol/L) (6). The source of this soluble FAP is unclear, although it may be shed from the plasma membrane through α2-antiplasmin (10). Therefore, FAP localization is not limited to the cell surface.

Some studies have revealed that systemic therapy targeting FAP^+^ cells can lead to severe cachexia, including muscle damage, osteotoxicity, and even death (11, 12). These findings raise concerns regarding strategies that target FAP and have largely hindered related research. Nevertheless, it is useful to identify treatments that selectively kill FAP^+^ locally activated fibroblasts, without causing systemic toxicity, which would permit treatments that aim to deplete tumor-associated FAP^+^ cells.

Previous reviews have summarized progress on the use of FAP in tumor diagnosis, however, many developments have been made since (13–15). There are also some studies focusing on FAP inhibitors and their radionuclides in tumors (16–18). This study focuses on the occurrence of FAP in tumors over the past ten years. It includes a comprehensive discussion of tumor development and updates on clinical applications, focusing on the progress of diagnosis and treatment of FAP tumors based on nanomaterials.

## Biological Properties of FAP

### Enzymatic Activity and Substrates

At the genetic level, human FAP and dipeptidyl peptidase-4 (DPPIV) genes share substantial homology. The FAP gene is located on chromosome 2q23 and contains 26 exons (total length: 73 kb), while DPPIV is located on chromosome 2q24.3 and contains 26 exons (total length: 70 kb). Therefore, some people believe that FAP evolved through the duplication of the DPPIV gene. Expression of FAP is observed in various animal species, including mice (19, 20) and xenopus (21), with the FAP gene in mice very similar to that in humans (located on chromosome 2, contains 26 exons, with a total length of 60 kb). Thus, mouse models are likely reliable for preclinical research regarding treatments that target FAP.

**Figure 1:**
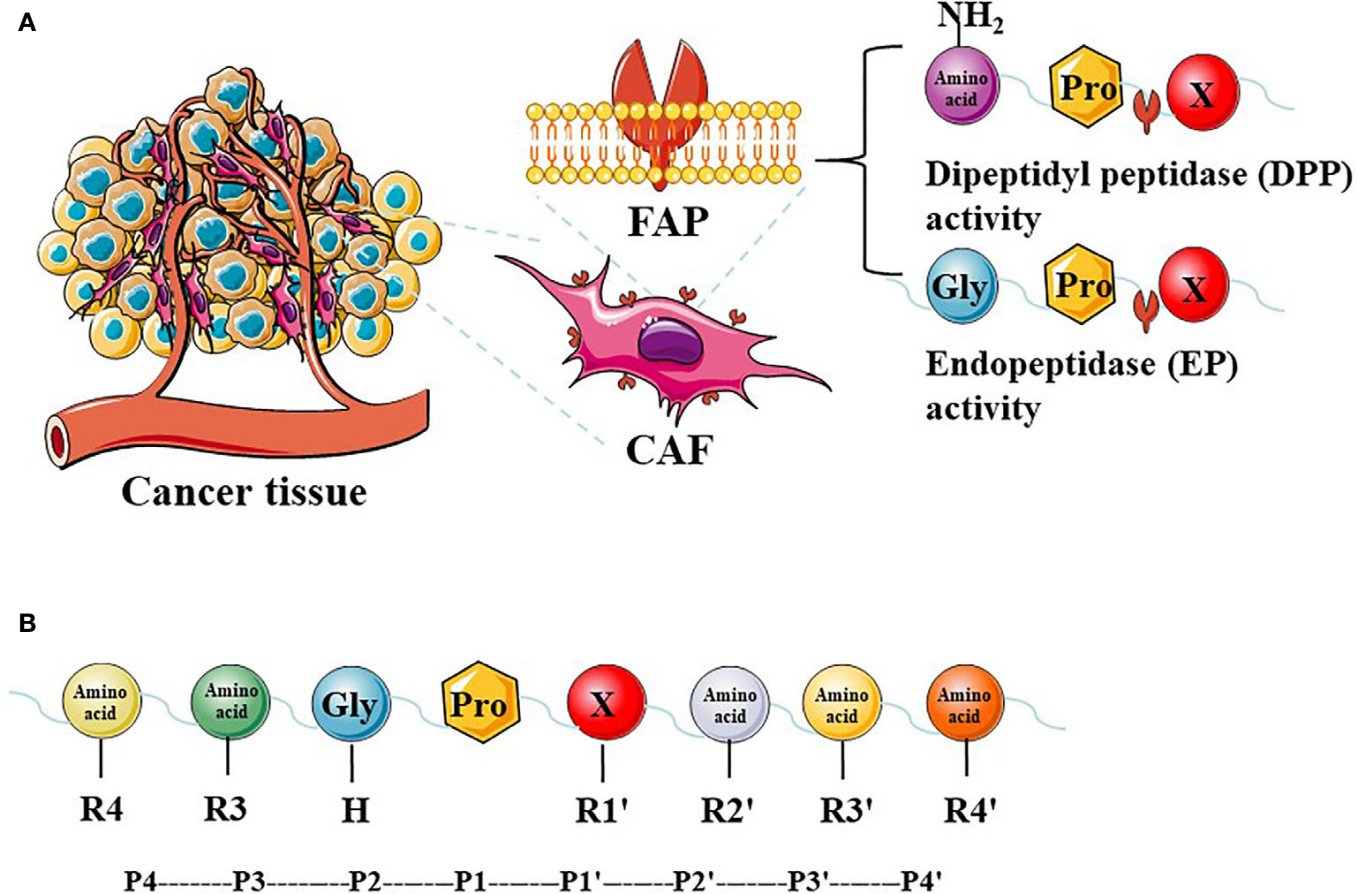
The expression of FAP in tumor tissues and FAP enzyme activity. Cancer-associated fibroblasts (CAFs) express high levels of fibroblast activation protein-α (FAP). FAP has dipeptidyl peptidase (DPP) and endopeptidase (EP) activity **(A)**. The P2 preferences depend on the type of enzymatic activity, and different amino acids at different positions have a greater impact on enzyme activity **(B)**.

The FAP protein is a 170-kda homodimer with two N-terminal glycosylated subunits. The 97-kda type II transmembrane serine protease is a member of the prolyl peptidase family, which includes DPPIV (most similar to FAP), DPP7, DPP8, DPP9, and prolyl carboxypeptidase. The FAP and DPPIV proteins have a 70% identity at the amino acid sequence (22) and share a catalytic triad of serine, aspartic acid, and histidine residues (23). Serine plays a nucleophilic role, which allows DPPIV to cleave the N-terminal Pro-“X” peptide bond (where “X” is any amino acid except proline or hydroxyproline). Unlike FAP, DPPIV is expressed in a variety of human tissues under normal conditions and is related to many physiological processes, including glucose homeostasis and T-cell activation (24). The FAP protein has dipeptidyl peptidase and endopeptidase activities, which are sometimes described as gelatinase activity. Although both FAP and DPPIV have dipeptidyl peptidase activity, the unique endopeptidase activity of FAP makes it preferentially cleave to the Gly-Pro-“X” sequence (**Figure 1A**), with the most effective cleavage when “X” is Phe or Met and the least effective one when “X” is His or Glu (25–27). In addition, this cleavage by FAP is impaired when the P4 and P2 residues are heavily charged amino acids (**Figure 1B**). Therefore, endopeptidase activity can be used to specifically detect FAP and are the basis of nanomaterial treatments that aim to specifically inhibit FAP.

Previous studies have shown that DPPIV can cleave neuropeptide Y, peptide YY, SP (Substance P), and brain natriuretic peptide 32, which can also be cleaved by FAP (28). The known active substrates of the FAP endopeptidase include collagens I, III, and V, as well as α-2 antiplasmin and fibroblast growth factor 21 (29). Other recently identified substrates of FAP include fibrillin-2, extracellular matrix protein 1, C-X-C motif chemokine 5, C1q, and tumor necrosis factor related protein 6 (C1qT6), and lysyl oxidase homolog 1 (**Table 1**). The ability of FAP to cleave collagen depends on previous matrix metalloproteinase activity or thermal degradation (30). Soluble FAP is known as α2-antiplasmin cleaving enzyme (APCE), which has pro-coagulation properties. After FAP cleaves α-2 antiplasmin, it is converted into a more effective plasmin inhibitor, which slows the dissolution of the fibrin clot and reduces bleeding during tissue repair (31).

**Table 1:** Differences between FAP and DPPIV on expression, enzyme activity, and natural substrates.

	Expression	Enzymatic activity	Natural substrate
FAP	Specific(fibrosis, arthritis, atherosclerosis, autoimmune diseases, metabolic diseases, and cancer)	EP & DPP	Collagens I, III and V; FBN-2; ECM-1;CCL-2, CXCL-5; C1qT6; LOX-L1;α-2 antiplasmin; NPY; PYY; SP; BNP; FGF21
DPPIV	Nonspecific	DPP	NPY; PYY; SP; BNP; FGF21

DPP, Dipeptidyl peptidase; EP, endopeptidase; FBN-2, fibrillin 2; ECM-1, extracellular matrix protein 1; CCL/CXCL, chemokines belonging to the CC and CXC family; C1qT6, complement C1q tumor necrosis factor-related protein 6; LOX-L1, lysyl oxidase-like-1; NPY, neuropeptide Y; PYY, peptide YY; SP, substance P; BNP, brain natriuretic peptide; FGF21, fibroblast growth factor 21.

### Non-Enzymatic Activity of FAP

Studies of mutated FAP with impaired enzymatic activity have revealed that FAP may have non-enzymatic functions. For example, transfection of mouse melanoma cell lines with non-enzymatically active FAP reduced their tumorigenicity. In contrast, FAP with normal enzymatic activity had enhanced tumorigenicity, which suggested that even low FAP enzymatic activity exerts biological effects (32). Similarly, breast cancer cell lines were transfected with a version of FAP that had low enzymatic activity exhibited faster tumor growth *in vivo* and faster degradation of the extracellular matrix (*vs*. untransfected cell lines) (33). Thus, FAP can induce tumor growth and extracellular matrix degradation, regardless of whether it has high or low enzymatic activity.

Another study of fibroblasts that were transfected with enzymatically inactive FAP revealed increased growth and migration of breast cancer cell lines, with FAP activating the phosphoinositide 3-kinases (PI3Ks), matrix metallopeptidase 2, and matrix metallopeptidase 9 signaling pathways (34). Other studies have indicated that FAP was highly expressed in oral squamous cell carcinoma cells, where FAP gene knockout inhibited tumor cell proliferation, migration, and invasion through the inhibition of the phosphatase and tensin homolog/PI3K/protein kinase B and Ras-ERK signaling pathways (35). Moreover, FAP can form complexes with DPPIV, matrix metallopeptidase 1, matrix metallopeptidase 2, urokinase-type plasminogen activator, and other proteins, which can act as inter-cell signal transduction pathways to promote tumor cell invasion (36, 37). The combination of FAP and integrin regulated downstream RhoA activity and influenced the migration of bone marrow-derived mesenchymal stromal cells, as the FAP protein loss significantly inhibited migration, although the peptidase activity of FAP did not play a role in this process (38). Inflammatory cytokines (IL-1β, TGF-β, and TNF-α) also promote the migration of bone marrow-derived mesenchymal stromal cells by upregulating FAP expression. Another study also revealed that FAP positively activated signal transducer and activator of transcription (STAT3) in fibroblasts through the urokinase receptor-dependent focal adhesion kinase-Src-Janus kinase 2 signaling pathway (39). The murine model of liver cancer indicated that FAP^+^ cancer-associated fibroblasts (CAFs) are the main source of chemokine (C-C motif) ligand 2 (CCL2), and fibroblast STAT3-CCL2 signaling inhibited tumor growth through enhanced recruitment of myeloid-derived suppressor cells. Moreover, FAP expression is positively correlated with levels of CCL2 and STAT3.

Cells that overexpress FAP have increased proliferation and migration, due to activation of the PI3K and sonic hedgehog pathways. Phosphorylation of FAP may be reduced when it forms a complex with the focal adhesion kinase protein, which may ultimately promote FAP overexpression. In this context, PI3K and sonic hedgehog inhibitors can inhibit FAP expression, which reduces cell proliferation and migration (36).

## Expression of FAP in Tumors

Normal tissues have low and generally undetectable levels of FAP expression. However, FAP is overexpressed in many tumor tissues, including breast (40–43), colorectal (44–46), pancreatic (47–50), lung (51–53), brain (54–56), intrahepatic bile duct (57), and ovarian (58–61) cancers. In addition, high levels of FAP expression can be detected in some tumors that are derived from non-epithelial tissues, such as melanoma (2, 62) and myeloma (63). In these tumors, FAP overexpression is typically observed in the interstitium, which has led to FAP being considered a universal marker for CAFs, although FAP expression can also be detected in gastric carcinoma (64–66), pancreatic carcinoma (67) and melanoma (2) cells.

## Roles of FAP in Tumors

**Figure 2:**
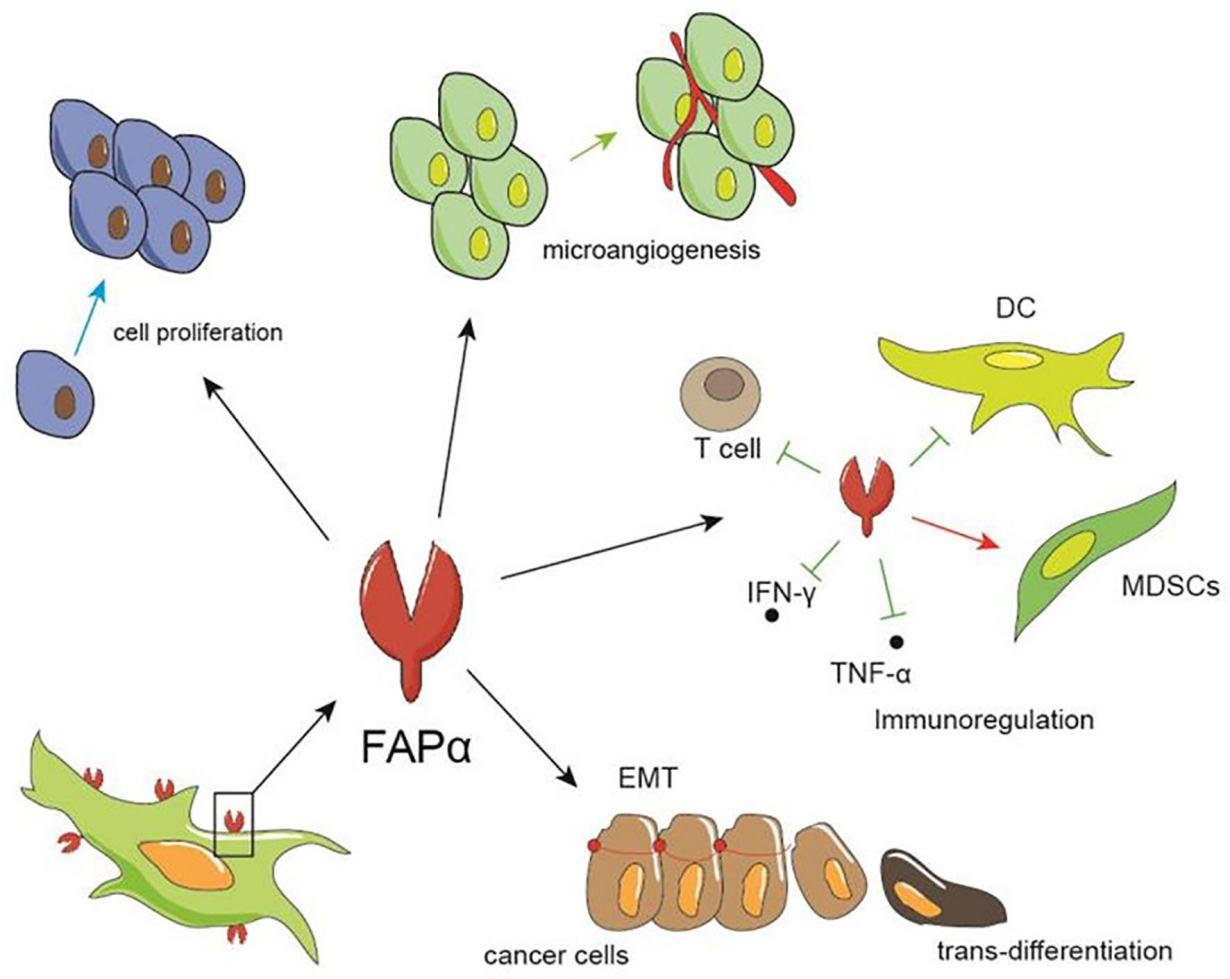
The role of FAP in tumors. FAP can promote tumor growth by facilitating the proliferation and invasion of tumor cells, promoting the formation of microvessels, and regulating immunity, such as inhibiting the differentiation and maturation of dendritic and T cells, thereby increasing the proportion of myeloid suppressor cell, and up-regulating IFN-γ and TNF-α.

The broad range of FAP expression in a variety of cancers has led to numerous studies regarding the pro-tumor and anti-tumor effects of FAP expression. The cumulative results of which indicate that FAP expression influences tumor growth by impacting tumor cell proliferation and invasion, angiogenesis, epithelial-to-mesenchymal transition, immunosuppression, and drug resistance (**Figure 2**).

### Promoting Tumor Cell Proliferation and Invasiveness

Numerous studies have indicated that FAP promotes tumor cell proliferation, migration, and invasion, which ultimately leads to tumor growth. There are two main hypotheses regarding the underlying mechanisms (3, 32–37, 68, 69). The first hypothesis involves an indirect mechanism, whereby FAP regulates extracellular matrix remodeling that leads to enhanced tumor growth and invasion (3, 32–34). It remains unclear whether FAP regulates this extracellular matrix remodeling through its enzymatic or non-enzymatic activity. The second hypothesis involves a direct mechanism, whereby FAP expression influences signaling pathways that control the cell cycle and proliferation, which ultimately promote tumor growth (35–37, 68–70).

The direct hypothesis is supported by many studies. In the indirect hypothesis, transfection of small interfering RNA targeting FAP inhibits the proliferation of ovarian CAFs, leading to cell cycle arrest (68). In squamous lung cancer cell lines, FAP overexpression promotes proliferation, migration, and invasion, accompanied by upregulation of the PI3K/protein kinase B and sonic hedgehog/glioma-associated oncogene signaling pathways (70). Other studies on oral squamous cell carcinoma have indicated that FAP is an upstream regulator of phosphatase and tensin homolog PI3K/protein kinase B and Ras-ERK signaling pathways (35). Additionally, Kawase et al. (69) studied the effects of co-culturing FAP^+^ fibroblasts with pancreatic ductal adenocarcinoma (PDAC) cell lines and reported increased phosphorylation of tumor suppressor genes in cancer cells leading to enhanced cell cycle progression and proliferation.

### Microangiogenesis

Aimes et al. (71) reported that human endothelial cells can produce FAP, which plays a regulatory role in microvascular reorganization and capillary morphological changes, in addition to other serine proteases. In this context, FAP^+^ breast cancer cells do not exhibit *in vitro* proliferation advantages, although inoculation into severe combined immunodeficiency mice leads to faster tumor growth and a higher degree of vascularization. Histological analysis of gastric cancer biopsy specimens also revealed that higher FAP expression is associated with a significantly greater density of microvessels (72). *In vivo* models of lung cancer and colon cancer also revealed that *FAP* knockout or drug-based inhibition are associated with decreased microvessel density and slower tumor growth (73). Another study revealed that FAP is expressed in human endothelial cells during the early stage of capillary formation, although FAP expression is absent in the mature endothelium (74). In addition, FAP expression is observed in endothelial cells at the invasive front of ductal carcinoma, suggesting that FAP promotes capillary growth and invasion of the extracellular matrix (75). Abundant FAP expression is also observed around abnormally proliferating blood vessels in the stroma of glioblastoma (54). A large number of studies have confirmed that FAP is expressed on the microvascular endothelial cells of malignant tumor tissues, such as multiple myeloma, gastric cancer, and breast cancer (63, 65, 76).

### Epithelial-to-Mesenchymal Transition

The epithelial-to-mesenchymal transition allows malignant epithelial cells to acquire a mesenchymal phenotype that permits increased migration and invasion required for metastasis (3). Studies have confirmed that antibodies targeting FAP can be used to isolate fibroblasts and reduce the purity of primary cells, and many epithelial-derived cell lines also express FAP during the epithelial-to-mesenchymal transition after transforming growth factor beta induction (77). Thus, although most FAP^+^ cells are derived from CAFs, some epithelial cells can express FAP under certain conditions.

### Immunomodulation

Studies have shown that FAP^+^ CAFs can promote an immunosuppressive tumor microenvironment by interfering with the differentiation and maturation of dendritic cells, blocking the conversion of T-cells to cytotoxic T-cells, and inhibiting the expression of the major histocompatibility complex antigens (78). One study revealed that relative to FAP^-^ CAFs, FAP^+^ CAFs have unique inflammatory gene expression characteristics, with the greatest upregulation of CCL2 expression (39). In addition, upregulation of CCL2 by FAP is not related to its enzymatic activity, as treatment using talabostat (an inhibitor of FAP enzymatic activity) does not alter CCL2 protein expression. The same research group investigated the role of FAP^+^ CAFs and mixed cells in the Hepa1-6 hepatoma cell line and observed that relative to FAP^-^ CAFs and mixed cells, the tumor model produced using the FAP^+^ CAFs mixture has higher proportions of polymorphonuclear myeloid-derived suppressor cells, myeloid-derived suppressor cells, and macrophages, and lower proportions of interferon gamma (IFNγ) IFNγ^+^CD8^+^ T-cells. Furthermore, in *CCL2* knockout mice, tumors with FAP^+^ CAFs have the same proportion of myeloid-derived suppressor cells as tumors with FAP^-^ CAFs, although the tumors lose their growth advantage. These results suggest that FAP^+^ CAFs release CCL2, which is recognized by the CCL2 receptor (CCR2) on circulating myeloid-derived suppressor cells and leads to their recruitment to tumor tissues (39).

Another colorectal cancer study confirmed that FAP^+^ CAFs produce CCL2 and exert similar effects on myeloid-derived suppressor cells. Feig et al. (67) speculated that FAP^+^ CAFs serves as the main source of tumor C-X-C motif chemokine 12 (CXCL12), which is involved in the local immunosuppressive environment. After adding inhibitors of the CXCL12 receptor C-X-C chemokine receptor type 4, the authors noted a T-cell-dependent reduction in tumor volume and improved response to anti-PD-1 treatment, although no clear anti-cytotoxic T-lymphocyte-associated protein-4 effects occur. Another study confirmed that FAP^+^ CAFs secrete CXCL12 after recognizing adenosine through the A2B adenosine receptor (79).

Kraman et al. (9) described the ability of FAP^+^ cells to suppress the anti-tumor immune response, which they evaluated using a transgenic mouse model in which the FAP gene was modified to include coding sequences for green fluorescent protein or diphtheria toxin receptor. They used the green fluorescent protein-expressing model to confirm that FAP was expressed in different CD45^+^ and CD45^-^ cells and noted that immunogenic tumors could be created through forced expression of ovalbumin. Further, prophylactic treatment of the mice with a vaccine successfully slowed tumor growth. The diphtheria toxin receptor-expressing model confirmed that diphtheria toxin reduced the number of FAP^+^ cells. When the ovalbumin vaccine was tested in the transgenic mice, the researchers observed that tumor growth stopped immediately after the diphtheria toxin-related decrease in FAP^+^ cells, although a similar result was not observed for the non-immunogenic tumors. Furthermore, the researchers confirmed that T-cell counts in the mice were not related to FAP expression, suggesting that FAP mediates the immune response through an alternate mechanism. Moreover, anti-tumor necrosis factor alpha and anti-IFNγ treatment counteracted the reduced tumor growth that was observed after the decrease in FAP^+^ cells, suggesting that FAP may inhibit the production of tumor necrosis factor alpha and IFNγ or weaken the cellular response to these cytokines. The cytokine levels also did not change in response to the decrease in FAP^+^ cells, which supports the latter hypothesis that FAP reduces the response of tumor cells to tumor necrosis factor alpha and IFNγ. The same group used diphtheria toxin receptor-expressing transgenic mice to study the role of FAP in PDAC and found that eliminating FAP^+^ cells significantly reduces tumor growth, which is related to CD4^+^/CD8^+^ T-cell activity. The decrease in FAP^+^ cells also enhances the response to anti-PD-1 and partially improves the efficacy of anti-cytotoxic T-lymphocyte-associated protein-4 treatment (67). Thus, in a murine model, FAP is involved in the resistance of PDAC to these immune checkpoint inhibitors. *In vivo* models of colorectal cancer also indicate that colorectal cancer cell lines co-injected with FAP^+^ CAFs have increased resistance to anti-PD-1 treatment (78), while *in vivo* models of gastric cancer revealed that anti-PD-1 treatment and FAP inhibitors have a synergistic effect in terms of slowing tumor growth (64). Therefore, it appears that FAP is involved in modulating the immune environment of tumors.

However, not all studies have shown that FAP exerts an immunosuppressive effect. For example, the elimination of CAFs can cause immunosuppression in pancreatic cancer, which in turn leads to shortened patient survival (80). Another study on non-small cell lung cancer revealed that in tumors with high CD3^+^/CD8^+^ T-cell infiltration, high FAP expression is associated with increased patient survival (81). These results suggest that FAP^+^ CAFs may have beneficial effects in some settings, and thus caution is warranted regarding treatments that aim to directly eliminate FAP^+^ CAFs.

## Targeting FAP

As described in the previous sections, FAP expression appears to be related to the occurrence and development of malignant tumors, and FAP expression appears to be highly specific to tumor tissues. Thus, relative to directly targeting cancer cells, there may be diagnostic and treatment benefits associated with targeting the FAP^+^ tumor stroma. Therefore, several research groups have explored strategies for treating various tumors by targeting either FAP itself or FAP^+^ CAFs (**Table 2**). Many tracer drugs that target FAP have shown great clinical promise, not only for diagnosis, but also for treatment (131).

**Table 2:** Summary of targeting FAP drugs.

	Drug	D or T	Nanodrug or not	EPR	Active position	mechanism	limitation	advantage	Ref.
**Immunotherapy**	FAP Vaccine	T	N	N	Targeting cancer cells &CAFs	Immunity therapy	Systemic toxicity	Activate autoimmune therapy for cancer	(82)
		T	N	N	Targeting FAP plasmid				(83)
		T	N	N	Targeting FAP DNA				(84)
	(CAR) T-cells	T	N	N	FAP^+^ cell				(12, 85–90)
**Inhibitor**	FAPI	D&T	N	N	FAP	Specific binding and inhibition of enzyme activity	The inhibitory effect cannot completely prevent tumor growth.	Radionuclide imaging has been applied to the human body.	(91–100)
**Based on antibodies**	F19	D&T	N	N	Specific antigen of FAP	Antigen and antibody specific binding	Low drug load	High specificity	(101, 102)
	Anti-scFv	D&T	N	N					(19, 103–105)
	OncoFAP,	D&T	N	N					(106)
	Sibrotuzumab	T	N	N			systemic toxicity	Clinical research	(107)
	ScFv-Z@FRT	D&T	Y (12.25 ± 1.51 nm)	Y	CAFs & ECM	PTT suppresses CXCL12 secretion and ECM deposition	The research is in the initial stage, the biological effect is not clear, and it is only in the animal experiment stage.	Enhance cytotoxic T cell infiltration	(108)
					CAFs & ECM	PTT destroy the dense tumor ECM		Improve tumor accumulation of nanoparticles	(109)
	Anti-FAP-IL	D	Y (139.1 ± 25nm)	Y	CAFs	Only FAP-expressing cells were able to take up and activate fluorescence of anti-FAP-IL *in vitro*		A valuable diagnostic tool for future stratification of patients before therapy	(110)
	PNP/siRNA/mAb	D&T	Y (10-60nm)	Y	CAFs	Specifically downregulated CXCL12 expression in CAF		Tumor metastasis inhibition	(111)
	F-SOS/DC/DOX	D&T	Y(50nm)	Y	CAFs & ECM	Light irradiation		Deep delivery	(112)
**Based on FAP Dipeptidase Activity**	Prodrug	T	N	N	CAFs & ECM	With a FAP-specific dipeptide (Z-Gly-Pro)	No passive targeting, low drug/fluorescence accumulation	The type of prodrug varies with the drug	(113–121)
	Fluorogenic probes	D	N	N	CAFs	The fluorescence quenched by the chemical environment of the fluorophore is recovered following enzymatic cleavage of the FAP-specific sequence.		The fluorescent cleavage product of such substrates enables quantification of FAP activity and thus quantity in solution and/or tissues.	(122–125)
	GNS@PDA	D&T	Y (90nm)	Y	CAFs & ECM	Incorporate FAP activatable NIR fluorescence imaging	The research is in the initial stage, the biological effect is not clear, and it is only in the animal experiment stage.	Multimodal imaging and PTT	(126)
	CAP-NPs	D&T	Y (80nm)	Y	CAFs & ECM	The CAP-NPs upon FAP cleavage resulted in rapid and efficient release of the encapsulated drugs specifically at tumor sites.		Disrupt the stromal barrier and enhance local drug accumulation	(127)
	Nanofibers	T	Y (5.2 ± 0.3nm)	Y	CAFs	The assembly/aggregation induced retention (AIR) effect endows the probe enhanced accumulation and retention around the tumor		5.5-fold signal enhancement in tumor compared to that of the non-aggregatable control molecule post 24 h administration through ex vivo imaging	(128)
	ZGD-MNs	T	Y (86.6nm)	Y	CAFs & ECM	Developed a nano-micelle system to facilitate the systemic delivery of Z-GP-Dox		Solved the insoluble nature of Z-GP-Dox & accumulative drug release was more than 56% within 24 h	(129)
	HA@DSP-pep-DSP	D&T	Y (10-80nm)	Y	CAFs & ECM	CAF-triggered transformable drug delivery system based on a cleavable peptide responsive to fibroblast activation protein-α (FAP-α)		Deplete the stromal barrier & Deeply penetrated the core region of the tumor	(130)

ECM, extracellular matrix; PTT, Photothermal therapy; NIR, near-infrared; D, Diagnosis; T, Treatment; Y, Yes; N, No.

### Inhibitor of FAP

Talabostat is a small molecule that inhibits the dipeptidyl peptidase activity shared by DPPIV and FAP. Preliminary findings revealed that oral talabostat treatment slows tumor growth in mouse models of fibrosarcoma, lymphoma, melanoma, and rhabdomyosarcoma, as well as in bladder cancer cell lines (91, 92). Talabostat also enhances the efficacy of oxaliplatin in mouse models of colon cancer (93). Thus, talabostat has been evaluated in various clinical trials, and a phase II trial revealed tumor control in 21% of patients with colorectal cancer (94). Although talabostat may be useful in this setting, additional studies are needed to identify strategies to improve its efficacy.

After years of research, the latest selective FAP inhibitor with low nanomolar potency was UAMC-1110. Meletta et al. (95) reported the first inhibitor-based probe of UAMC-1110 in 2015, which was originally designed to visualize atherosclerotic plaque. However, *in vitro* studies have found that this method is not useful for the expected atherosclerosis imaging, but it seems to be highly correlated with tumor tissue imaging.

The same inhibitory drugs are also used to study nervous system tumors. One preclinical study revealed that fibroblast activation protein inhibitor-04 (FAPI-04) exhibits great tumor accumulation and delayed elimination. In addition, FAP-specific positron emission tomography revealed increased tracer uptake in glioblastoma and high-grade mutant astrocytoma, without significant uptake in diffuse astrocytoma (96). Loktev et al. (97) used inhibitor-based radiopharmaceuticals to selectively target a variety of tumors with high FAP expression and designed several fibroblast activation protein inhibitor variants to further increase tumor accumulation and the tracer effect, which improved the therapeutic effect. Moreover, Watabe et al. (98) used ^64^Cu and ^225^Ac to radioactively label fibroblast activation protein inhibitor-04, permitting live tracking and treatment response evaluation for pancreatic tumors that were transplanted into an *in vivo* mouse model. Recently, 68Ga-FAPI has been clinically adopted, allowing researchers to obtain a variety of tumor images with very high uptake and image contrast, paving the way for new applications in tumor characterization, staging, and treatment. Qin et al. (99) evaluated the performance of 68Ga-DOTA-FAPI-04 (68Ga-FAPI) PET/MR in the diagnosis of primary tumors and metastatic lesions in patients with gastric cancer and compared it with 18F-FDG PET/CT. The results show that, due to the high expression of FAP in gastric cancer and its metastatic tissues, 68Ga-FAPI PET/MR is significantly superior to 18F-FDG PET/CT for the diagnosis of primary gastric cancer and its metastatic lesions. Thus, 68Ga-FAPI PET/MR may represent a promising diagnostic method that is expected to replace 18F-FDG PET/CT in the future.

Recently, a detailed preclinical study evaluated the role of the highly selective FAP inhibitor UAMC-1110 in a mouse model of pancreatic cancer. UAMC-1110 did not slow down tumor growth, nor did it enhance the effect of radiotherapy. According to existing data, inhibiting FAP enzyme activity may have some beneficial effects on the tumor microenvironment, but it may not be sufficient to prevent tumor progression (100). Instead, the super binding ability of FAP inhibitors has been widely used in tumor diagnosis.

### Immunotherapy Targeting FAP

#### FAP Vaccine

Chen et al. (82) evaluated a whole-cell tumor vaccine targeting FAP, which suppressed tumor growth by simultaneously attacking cancer cells and CAFs. Subsequent studies evaluated heterologous antigens to improve whole-cell tumor vaccines by eliminating immune tolerance and activating the adaptive immune response. One study revealed that this led to an increased number of apoptotic tumor cells and decreased number of CAFs, which was associated with delayed tumor growth and lower recurrence. Additional experiments revealed that the anti-tumor response was related to antigen-specific cytotoxic T-cells, as well as activation of the humoral immune response. Immunized mice produce antibodies to FAP, which can be detected in their serum, and this FAP-based heterogeneous whole-cell tumor vaccine treatment is a potential strategy for personalized immunotherapy in cancer patients. Wen et al. (83) used cationic liposomes to encapsulate a FAP plasmid, and this vaccine was able to inhibit tumor growth and metastasis in a mouse model of colon cancer. Loeffler et al. (84) constructed an oral vaccine targeting FAP DNA, which was used to pre-treat mice that were subsequently injected with colon cancer or breast cancer cells. The oral vaccine was associated with reduced tumor growth, suppression of lung metastasis, increased chemotherapy uptake, and increased survival that was related to a CD8^+^ T-cell-dependent mechanism. Animal models have also confirmed that the vaccine increased T-cell activation, with one-half of the immunized mice not developing tumors after being injected with tumor cells, while those that did develop tumors had significantly prolonged survival.

### Chimeric Antigen Receptor T-Cell Therapy Targeting FAP

Chimeric antigen receptor (CAR) T-cells are an exciting new immunotherapy strategy, using cytotoxic T-cells that are artificially targeted to recognize specific antigens and thus eliminate cancer cells (85–87). The US Food and Drug Administration has approved CAR T-cell therapy for certain forms of leukemia and lymphoma. *In vivo* studies have s-oma, mesothelioma, breast cancer, colon adenocarcinoma, and lung adenocarcinoma (88). Schuberth et al. (89) also demonstrated that FAP expression existed in all subtypes of malignant pleural mesothelioma, and that CD8^+^ T-cells targeting FAP had strong therapeutic potential *in vitro* and *in vivo*, based on reduced FAP^+^ tumor growth and improved survival among mice in the FAP^+^ model. However, FAP expression in malignant cells is limited to a few cancer types. Thus, targeting FAP^+^ stromal cells with CAR T-cells can greatly broaden the application of this therapy, and we hypothesize that using CAR T-cells to selectively eliminate FAP^+^ cells may improve patient survival, given the tumor-promoting effects of FAP^+^ CAFs. Kakarla et al. (90) have demonstrated that CAR T-cells can effectively kill FAP^+^ cells *in vitro* and increase overall survival in a mouse model of lung adenocarcinoma.

Despite these benefits, caution is warranted regarding the clinical application of CAR T-cells targeting FAP. For example, one study revealed that FAP-specific CAR T-cells did not regulate tumor growth and might instead induce lethal osteotoxicity and cachexia by killing pluripotent stem cells in the bone marrow stroma (12). Although the cause of this serious adverse effect is unclear, it may be related to differences in FAP specificity, which suggests that further research is needed to optimize CAR T-cell therapy targeting FAP.

### Nanodrugs Targeting FAP for Diagnosing and Treating Tumors

Nanotechnology is an emerging field that aims to evaluate and modify natural processes on a nanometer scale. Nanomaterials have special properties that produce quantum size, interface, and macroscopic quantum tunneling effects, which can induce different biological responses. For example, the ultra-small size and relatively large surface area of nanoparticles make them effective for drug loading, as the nanoparticles can easily penetrate blood vessels without causing vascular endothelial damage and with a limited risk of enzymatic degradation. Furthermore, the local drug concentration is high, which can improve the curative effect and reduce systemic side effects. Moreover, nanoparticles have high surface reaction activity, many active centers, high catalytic efficiency, and strong adsorption capacity, which may have broad applications in the diagnosis and treatment of tumors. Some fluorescently labeled nanomaterials also have a significant role in tumor diagnosis and treatment, particularly those emitting second near-infrared channel (NIR-II) region fluorescence. Fluorescence imaging in the NIR-II region is at the forefront of biomedical research due to its inherent advantages, including relatively lower tissue autofluorescence and higher spatiotemporal resolution (132–134). Additionally, certain nanomaterials are constructed out of materials that respond to enzyme activity, and consequently, turn on quenched fluorescence under the catalysis of enzymes to obtain clear images (135).

### Targeted Drugs and Nanodrugs Based on Antibodies to FAP

Early studies of monoclonal antibodies targeting FAP focused on identifying FAP and F19 without considering their potential therapeutic effects. However, radiolabeled F19 revealed high expression of FAP in tumors and metastases, with improved clinical symptoms, which supports potential diagnostic and therapeutic roles for antibodies targeting FAP (101, 102). Cheng et al. (19) reported that antibody treatment targeting FAP inhibits tumor growth. Specifically, they immunized rabbits with recombinant mouse FAP and collected the resulting serum with FAP-specific antibodies. Treatment with this serum significantly inhibited the growth of colorectal cancer cell lines transplanted into nude mice. Specific anti-FAP antibodies and single-chain variable fragments (scFv) against FAP were subsequently developed, and the results revealed that the human single-chain fragments (scFv18 and scFv34) have greater affinity and lower immunogenicity, relative to F19 (103–105).

As macromolecular antibodies cannot easily enter solid tumors and produce a curative effect, Schmidt et al. constructed a bivalent FAP-specific antibody through targeted selection and reported an increased affinity for tumor tissue and human-derived V_L_ and V_H_ chains (104). Results from a phase I clinical study and pharmacokinetic analysis of the humanized anti-FAP antibody (sibrotuzumab) further revealed that it is well tolerated in humans and is specifically concentrated in the tumor stroma, with limited absorption in normal tissues (107). Moreover, Millul et al. (106). described a ligand with ultra-high affinity for FAP (OncoFAP) that is used for precise diagnosis and treatment of FAP^+^ tumors. Through the addition of fluorescein, which facilitates quantification of drug aggregation, they observed that 10 minutes after intravenous injection of OncoFAP, more than 30% of the drug had accumulated in 1 g of the tumor, maintaining a high concentration for at least 3 h, ensuring a long diagnostic time window. Furthermore, certain drugs can be tagged with fluorescein, allowing for simultaneous treatment and diagnosis.

Ruger et al. (110) combined liposomes containing DY-676-COOH with antibodies of FAP (scFv) to prepare anti-FAP liposomes (anti-FAP-IL). After the synthesis, the fluorescence of near-infrared fluorescent dyes in the aqueous solution within the liposomes was quenched, and Only FAP-expressing cells were able to take up and activate fluorescence, which improves the diagnostic accuracy of the tumor. Li et al. (109) reported a nanoparticle-based photodynamic therapy involving a photosensitizer (ZnF16Pc) encapsulated in a ferritin nanocage. The nanocage was conjugated with scFv to permit FAP targeting, and phototherapy was then used to eliminate the targeted cells. Targeting CAFs effectively treated tumors in mice. An analysis of intratumoral aggregation at different nanoparticle sizes revealed that nanoparticles in the tumors had enhanced permeability and retention, although most nanoparticles were distributed in the area around blood vessels and did not migrate to the tumor’s center. This was largely related to the physical barrier that was created by the dense tumor extracellular matrix, which prevented effective nanoparticle extravasation and diffusion. Thus, targeting and eliminating CAFs promotes the aggregation of nanoparticles in the tumors. Zhen et al. (108) evaluated a similar nano-photoimmunotherapy strategy using drug-bearing nanocages conjugated with scFv to target FAP, which effectively eliminated CAFs through phototherapy with negligible damage to healthy tissues. Although this method did not directly target cancer cells, it effectively reduced tumor growth in tumor-bearing mice. Further studies revealed that targeting CAFs leads to decreased CXCL12 release and decreased extracellular matrix deposition, which reduces the barrier to T-cell infiltration and facilitates greater tumor inhibition. Thus, targeting FAP in this context might help regulate the tumor microenvironment and permit a more effective anti-tumor immune response. Lang et al. (111) built a CAF-targeted siRNA delivery system by loading FAP antibodies onto cell-penetrating peptide (CPP)-based nanoparticles. The nanoparticles can specifically down-regulate the expression of CXCL12 in CAFs, significantly inhibiting tumor cell invasion and migration, and tumor angiogenesis.

Due to the special morphology of nanomaterials, more antibodies are attached to the surface, which improves the possibility of binding to FAP and greatly enhances the targeting ability.

### Prodrugs and Nanodrugs Based on FAP Dipeptidase Activity

**Figure 3:**
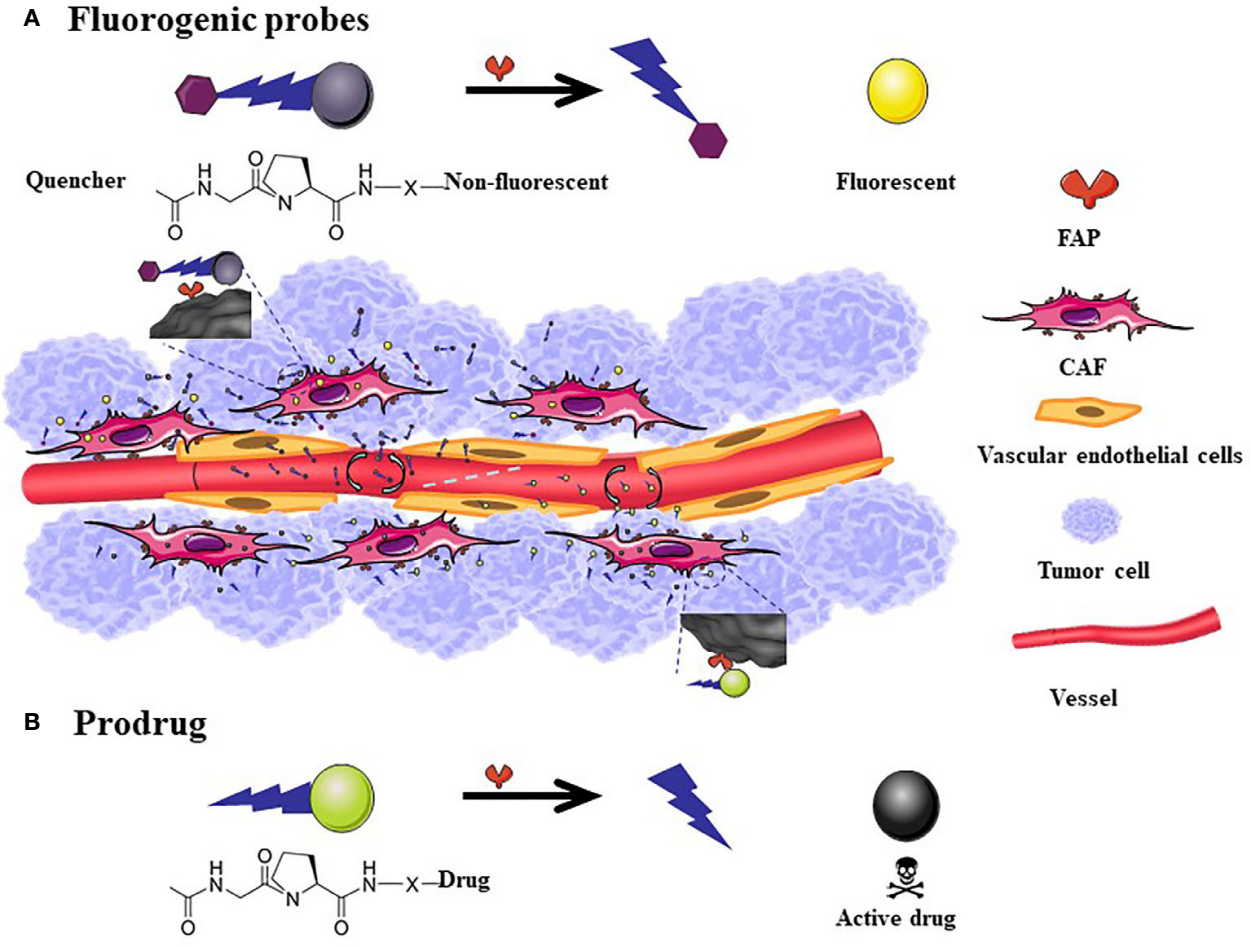
The mechanism of action of fluorogenic probe and prodrugs. The general scheme for Fluorogenic probes is that the quenched fluorescence is restored after the FAP-specific peptide is cleaved **(A)**. FAP targeted prodrugs: X indicates variable linkage between cleavable bond and drug in the C-terminal part of the prodrug. The toxicity of the drug reappears after the prodrug is cleaved **(B)**. The size of fluorescent probes and prodrugs is much smaller than the nanometer level. Although it can easily penetrate into tumor tissues, a considerable amount of drugs will flow back into the blood vessels, therefore, there is no passive targeting effect.

The general structure of the prodrug is Z-Gly-Pro-Drug (113–119, 129). It has a small molecular weight, and its toxicity is lower than that of the original drug. It exerts its effect after hydrolysis of the FAP-positive site (**Figure 3**).

#### Diagnosis

Based on the high expression characteristics of FAP in tumor tissues, fluorescent probes (122–124) (**Figure 3A**) and combined prodrug probes (125) that respond to FAP enzyme activity have been designed to diagnose tumors. The use of the above probes has been only tested in animal tumor models for the time being.

Han et al. (126) developed a nanodrug with FAP endonuclease as a switch based on polydopamine-coated gold nanostar (GNS@PDA). This nanoplatform can essentially perform computed tomography/photoacoustic/two-photon luminescence/infrared thermal four-modality imaging. Under the precise guidance of multi-mode imaging, GNS@PDA performs uniform photothermal ablation of large solid tumors. These results show the great potential of this scalable nanoplatform related to FAP in cancer treatment. Ji et al. (127) developed nanocarriers containing FAP cleavable peptides. These particles can hold drugs or fluorescent dyes and can release loaded drugs and fluorescent dyes under the action of FAP endonuclease, so they can be used as drug delivery platforms and cancer tissue imaging tools. Due to the high loading rate of nanodrugs, in most cases, integrated diagnosis and treatment research will likely be carried out, which can achieve the effect of killing two birds with one stone.

Zhao et al. (128) constructed a near-infrared probe based on FAP reactive peptides, which spontaneously form a large quantity of nanofibers on the surface of CAFs. *In vitro* imaging revealed that tumors were detectable at 24 h after probe administration and that the tumor group had a 5.5-fold greater signal than the control group. The probe provides a window of > 48 h for detecting a tumor and the selective probe assembly permits differentiation between tumors and organs with high metabolic activity, as the probe produces a tumor-specific signal that is 4-fold greater than the liver signal and 5-fold greater than the kidney signal. Moreover, the probe could specifically, and sensitively, diagnose small tumors with a diameter of approximately 2 mm.

#### Therapies

Considering that FAP is overexpressed in the tumor microenvironment and generally absent in healthy adult tissues, some research groups have sought to use FAP protease activity to selectively activate prodrugs at tumor sites to improve effectiveness and reduce toxicity. Most candidates are prodrugs that are modified using nanotechnology, although they have not been tested in clinical trials. A mouse breast cancer model revealed that epirubicin conjugated with a FAP-specific dipeptide (Z-Gly-Pro) effectively releases epirubicin after incubation with FAP, and epirubicin induces a substantial anti-tumor effect in cells with high FAP expression (4T1) (**Figure 3B**). Furthermore, relative to free epirubicin, mice treated with this nanomaterial exhibit less weight loss with no obvious cardiotoxic effects (120). Other mouse and dog models have revealed that doxorubicin conjugated to a FAP substrate has significantly lower toxicity and greater safety relative to the toxic effects of free doxorubicin on the heart, liver, kidneys, spleen, and peripheral blood leukocytes. Moreover, the same dose of the doxorubicin-conjugated formulation is associated with a 2-fold increase in intratumor accumulation. In the clinical trial stage, it was found that Z-Gly-Pro-Dox is difficult to dissolve in water (117). Zhang et al. (129) designed a nanomicelle system (ZGD-MNs) to promote the systemic administration of Z-Gly-Pro-Dox. A physiologically based pharmacokinetic model was used to evaluate its distribution in rats. The study found that ZGD-MNs are reasonably stable in phosphate buffer, showing good physical and chemical stability during the observation period of 2 weeks, and the cumulative drug release rate within 24 h was over 56%. Ji et al. (127) have designed a new cleavable amphiphilic peptide that specifically responds to FAP on the surface of CAFs. The peptide spontaneously assembles into fibrous nanostructures in solution, which can easily be converted into drug-loaded spherical nanoparticles. These nanoparticles break down in response to FAP activity, resulting in rapid and effective drug release at the tumor site.

### Deep Delivery of Nanodrugs in Tumor Tissues

**Figure 4:**
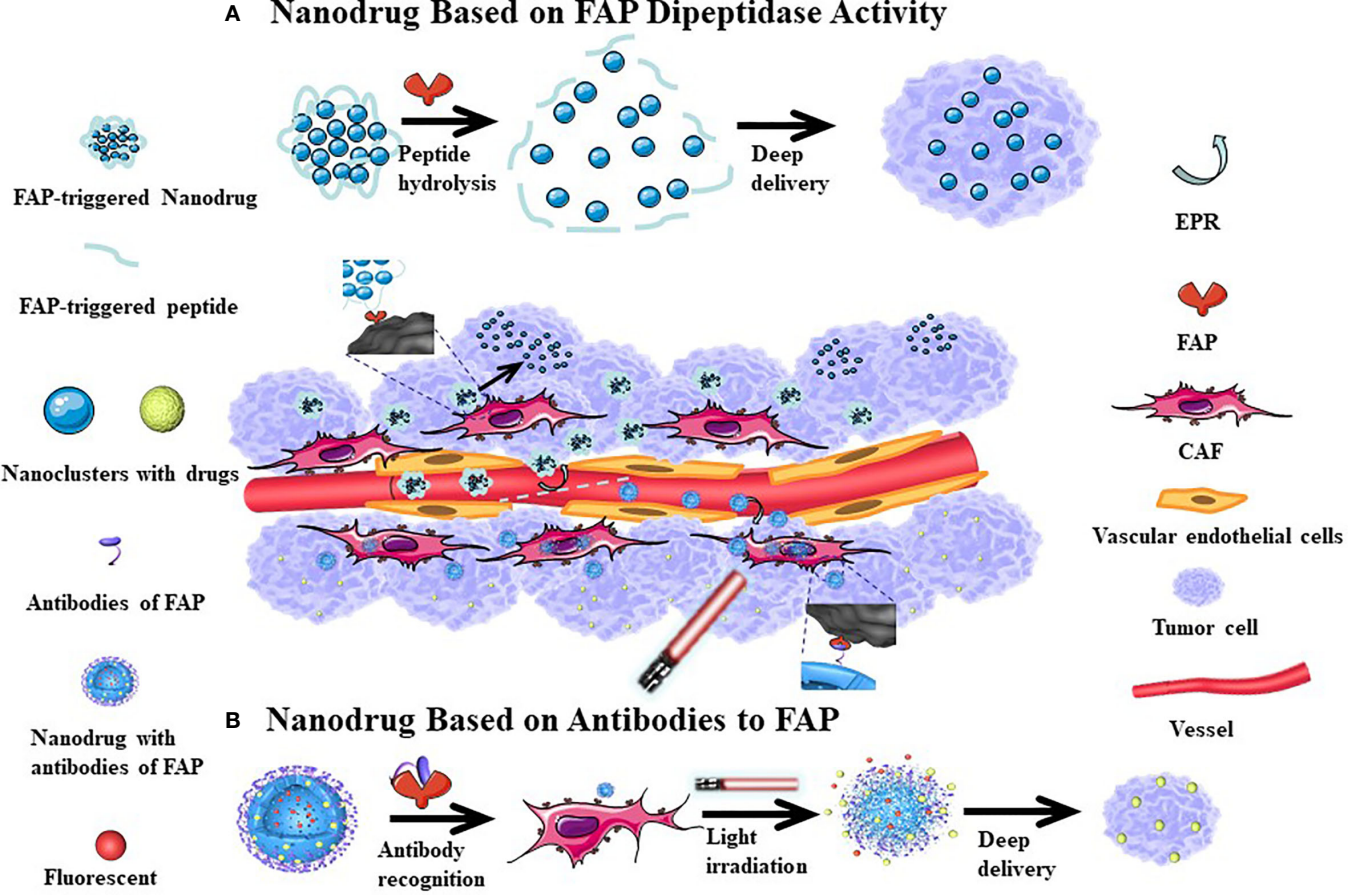
The mechanism of nanodrugs deep delivery in tumor tissues. After the cleavable peptide reacts with FAP, the nanoparticles are broken down into smaller nanoclusters which increases their dispersion **(A)**. More FAP antibodies can be be more easily attached to the surface of nanomedicine, which can increase the accumulation of drugs in tumor tissues. Following Light irradiation, the nanoparticle splits and the tumor tissue is fluorescently displayed, where at the same time, a part of CAFs is killed, with the small-sized nanoparticles continuing to have a deep tissue effect **(B)**. Since the tumor tissue vascular endothelial gap is relatively large, nano-sized drugs can enter the tumor tissue through incomplete vascular endothelium, with relatively few nano-drugs returning from the tumor tissue to achieve the EPR effect. This passive targeting effect causes nanomedicine accumulations in tumor tissues.

The performance of many anti-cancer drugs is largely hindered by insufficient penetration. However, the variability of the particle size in nanomedicine allows for better dispersion and infiltration after entering the tumor tissue. In addition, targeting FAP to induce CAFs damage can also increase the penetration depth of the drug in the tumor tissue. Hou et al. (130) have proposed a self-assembling FAP-triggered drug delivery system composed of peptide-crosslinked cationic polyaminoamine dendrimers. The chemotherapeutic drug (docetaxel) is conjugated to the peptide-crosslinked cationic polyaminoamine through disulfide bonds and electrostatic interactions and also coupled to hyaluronic acid to improve tumor targeting and biocompatibility. The nanoparticles have a diameter of approximately 200 nm and negative zeta potential, which permits stable circulation in the blood. However, when exposed to FAP, the nanoparticles dissociate and release the chemotherapeutic drug, which can penetrate CAFs and tumor cells. Studies have confirmed that the nanomaterial has good penetration of tumor-related biological barriers while killing a large number of tumor cells and a smaller number of CAFs. This effect is associated with a good therapeutic effect in hyperplastic solid tumors in connective tissues (**Figure 4A**). Yan et al. (112) have previously designed a light-triggered large-size nanoparticle. The FAP-α-targeting peptide was modified on the surface to increase the targeting of tumor tissue. After the photodynamic reaction, the large particles decomposed into small nanoclusters, which enhances drug delivery (**Figure 4B**). The photodynamic response simultaneously induces CAFs apoptosis and breaks the physical barrier that affects deep tumor delivery. Another study revealed that CAFs are closely associated with local angiogenesis while targeting FAP^+^ CAFs and administration of vascular disrupting agents that kill perivascular cells are associated with a less stable blood-tumor barrier and greater killing of tumor cells. Chen et al. (121) examined how this mechanism used vinblastine combined with a FAP substrate, which significantly reduced the growth of HepG2, A549, HeLa, and CNE-2 xenograft tumors. Both of these treatments rely on drug delivery through dipeptidase activity, which can kill CAFs and weaken the extracellular matrix, subsequently enhancing local drug accumulation. These strategies have good tumor specificity and therapeutic effects in a variety of solid tumor models.

## Conclusion and Future Direction

FAP is a marker that is constitutively expressed on the mesenchymal cells of most epithelial solid tumors. There is increasing understanding that expression of FAP promotes tumor occurrence, development, invasion, and metastasis, which worsens the patient’s condition and is associated with a poor prognosis. Furthermore, our understanding of the physiological effects of FAP expression has expanded to include its effects on the activation of tumorigenic signals, angiogenesis, the epithelial-to-mesenchymal transition, and even immunosuppressive functions. Thus, there is interest in FAP as a potential target for anti-tumor treatments, and existing research suggests that FAP-targeted drugs can exert curative effects in models of most solid tumors. Although some drugs have been evaluated in clinical studies, drug instability and systemic side effects have limited their application. Moreover, the complex interactions between the tumor microenvironment components have made it difficult to precisely determine the specific contribution(s) of FAP to tumor development. Although its clinical application is limited, nanotechnology is a promising field for addressing these issues by increasing drug delivery, solubility, and adsorption, which may promote greater tumor permeability and retention. Another option is targeted therapy, although there are limited data regarding the anti-cancer efficacy of drugs that solely target CAFs. Further research is needed to determine whether these drugs should be combined with chemotherapy, radiotherapy, targeted therapy, or even immunotherapy, as some researchers believe that simply eliminating CAFs might promote metastasis by de-stabilizing the extracellular matrix surrounding the tumor. Therefore, substantial work is needed to continue advancing our understanding of treatments targeting FAP and their anti-tumor effects. Building prodrugs on the basis of nano-platforms has absolute advantages in both fluorescence imaging capabilities and drug loading; coupled with its passive targeting effect (enhanced permeability and retention effect, EPR) (136–139) and deep delivery to tumor tissues, both of these greatly increase the accumulation of drugs in tumor tissues and reduce the concentration of prodrugs in non-target organs in the body. The structure of nanomaterials is relatively stable, reducing the possibility of accidental release in the circulation. To date, most nanomedicine remains in the research phase. The preparation of most nanodrugs is complicated while the synthesis conditions are strict, therefore, nanomedicine cannot be mass-produced. Furthermore, most nanodrugs used in medicine use materials with good biocompatibility, such as proteins and peptide chains. These materials are extremely easily degraded in the body and, even if they show good results *in vitro*, reactions may be slowed down in the complex environment of the organism, and some side effects may occur. Therefore, although nanomaterials have many advantages, there remain many unknown parameters that require further study, but we believe that nanomedicine will significantly improve disease treatment.

## Author Contributions

LX and JG drafted the manuscript. ZZhe, SL, YC, ZZha, and CY participated in the data review and collection for the study. RZ and XY conceived the study and participated in its design and coordination. All authors contributed to the article and approved the submitted version.

## Funding

This work has been financially supported by the National Natural Science Foundation of China [Nos. 82071987, 81771907]; Science and technology innovation team project of Shanxi Province [No. 201705D131026]; Engineering Technology Research Center of Shanxi Province [No. 201805D121008]; Scientific and Technological Achievements Transformation Project of Shanxi Province [No. 201704D131006]; Laboratory Construction Project of Shanxi Province, and the Projects for Local Science and Technology Development Guided by the Central Committee [No. YDZX20191400002537]; Research Project Supported by Shanxi Scholarship Council of China [No. 2020-177]; Fund Program for the Scientific Activities of Selected Returned Overseas Professionals in Shanxi Province [NO: 20200006]; and the applied basic research project of Shanxi Science and Technology Department under Grant [No. 201901D111416].

## Conflict of Interest

The authors declare that the research was conducted in the absence of any commercial or financial relationships that could be construed as a potential conflict of interest.

## Publisher’s Note

All claims expressed in this article are solely those of the authors and do not necessarily represent those of their affiliated organizations, or those of the publisher, the editors and the reviewers. Any product that may be evaluated in this article, or claim that may be made by its manufacturer, is not guaranteed or endorsed by the publisher.
